# Hospital Pharmacists’ Perspectives on Documenting and Classifying Pharmaceutical Interventions: A Nationwide Validation Study in Portugal

**DOI:** 10.3390/pharmacy13060159

**Published:** 2025-11-01

**Authors:** Sara Machado, Fátima Falcão, Afonso Miguel Cavaco

**Affiliations:** 1 Departamento de Farmácia, Farmacologia e Tecnologias de Saúde, Universidade de Lisboa Faculdade de Farmacia, 1649-003 Lisboa, Portugal; mfpfalcao@gmail.com (F.F.); acavaco@ff.ulisboa.pt (A.M.C.); 2 Pharmacy Department, Unidade Local de Saúde do Baixo Alentejo, 7801-849 Beja, Portugal

**Keywords:** pharmacist interventions, confirmatory factor analysis, hospital pharmacy

## Abstract

Pharmacist interventions (PIs) are central to optimising pharmacotherapy, preventing drug-related problems, and improving patient outcomes. In Portugal, the absence of a validated tool to consistently document and classify PIs limits data comparability and service development. Given these gaps, this study aimed to describe hospital pharmacists’ attitudes towards PI documentation and classification, following confirmatory factor analysis (CFA) of a survey instrument, and to provide a comprehensive overview of current practices and behaviours in hospital settings across Portugal. An online questionnaire, previously validated, was distributed online to all hospital pharmacists registered with the Portuguese Pharmaceutical Society (October–December 2024). Sociodemographic data and the cognitive and behavioural domains of pharmacists’ attitudinal model were analysed descriptively, and CFA tested the three-factor structure (Process, Outcome, Satisfaction) of the attitudinal affective domain. Of 1848 pharmacists, 260 responded (14%). Respondents reported performing a mean of 49 PIs/month (SD = 196), although many never recorded (28.8%), classified (56.2%), or analysed (52.3%) interventions. Only 2.7% declared to use a validated classification framework. The CFA supported the structural coherence of the Process factor but revealed some overlapping between Process and Outcome and instability in the Satisfaction factor. The nationwide scope and application of CFA provided partial support for the hypothesised model and highlighted areas for refinement, including revision of Satisfaction items and reconsideration of Process and Outcome as overlapping constructs. Findings highlight strong professional commitment to PIs but persistent barriers, including less clear procedures and satisfaction, underscoring the need for a unified, standardised national system to support consistent recording, classification, and evaluation.

## 1. Introduction

Pharmacist interventions (PIs) are a cornerstone of modern pharmaceutical care, playing a pivotal role in optimising pharmacotherapy, preventing drug-related problems (DRPs), and improving patient outcomes [1,2,3]. As healthcare systems increasingly prioritise safer and more effective medication use, the documentation and classification of these interventions have gained growing relevance, not only to support clinical decision-making, but also to demonstrate the impact of pharmacists within multidisciplinary teams [4,5].

Despite efforts to structure and harmonise the recording of PIs, many healthcare systems, such as Portugal’s, still lack validated tools capable of consistently documenting and classifying PIs. This gap poses risks to patient safety, as undocumented or inconsistently reported interventions hinder continuity of care and the prevention of DRPs [6]. It also limits data comparability, quality assurance, and service development, underscoring the need for robust, contextually appropriate instruments [7,8,9]. National frameworks such as Act-IP (France), GSASA (Switzerland), and Be-CLIPSS (Belgium) have demonstrated how standardised documentation systems can mitigate these risks by supporting consistent data collection, facilitating evaluation, and informing institutional and national strategies [10,11,12]. Understanding pharmacists’ behaviours, attitudes, and beliefs regarding PI documentation is essential to overcoming these limitations and fostering the institutional integration of pharmaceutical care.

To address this need, a questionnaire was recently developed and validated to assess hospital pharmacists’ attitudes and behaviours concerning the documentation and classification of PIs in Portugal [13]. Grounded in the tripartite model of attitudes, comprising affective, behavioural, and cognitive domains, the tool was designed to inform both practice and policy by capturing the psychological and operational dimensions that influence pharmacists’ engagement with both PI registration and classification. An exploratory factor analysis (EFA) was earlier conducted and identified the latent structure of the measurement scale, and the final questionnaire demonstrated acceptable psychometric properties, including reliability and validity, for assessing Portuguese hospital pharmacists’ involvement in PIs [13].

However, beyond identifying individual constructs, it is also important to examine the correlations among sub-dimensions. Therefore, a confirmatory factorial analysis (CFA) was employed to test the model’s dimensional structure further and assess its psychometric adequacy [14]. Confirmatory research serves multiple purposes, such as developing new measurement tools, assessing the psychometric characteristics of recently created instruments, validating theoretical constructs, and exploring the impact of measurement instruments [15].

Given the limited existing data on Portuguese pharmacists’ perspectives and practices in documenting and classifying PIs in hospital settings, it was also necessary to test the instrument in a larger, nationwide sample. Thus, the present study aims to describe hospital pharmacists’ attitudes towards PI documentation and classification, following CFA of the survey instrument, and to provide a comprehensive overview of current practices and behaviours in hospital settings across Portugal.

## 2. Materials and Methods

### 2.1. Participants and Procedure

The questionnaire was administered online via the QuestionPro© platform, which enabled secure questionnaire design and data storage, ensuring efficient survey management. The survey was distributed to all hospital pharmacists registered with the Portuguese Pharmaceutical Society, a mandatory requirement for professional practice in Portugal [16].

Participation was voluntary and anonymous, and informed consent was obtained electronically prior to the start of the questionnaire. The email invitation included a link to the survey and a consent form outlining the study objectives, ethical approval, confidentiality, and data protection procedures. The data collection period extended from 25 October to 31 December 2024, during which two reminder emails were sent. To avoid duplicate entries, each submission was tied to a unique IP address; duplicate, incomplete, or blank responses were excluded from the analysis, resulting in the removal of 16 responses.

### 2.2. Measures and Instruments

The instrument included 37 items, addressing pharmacists’ feelings, beliefs, and behaviours of hospital pharmacists regarding PIs in daily practice. The questionnaire was initially developed in Portuguese (an English translation is provided as Supplementary Materials). It consisted of an introductory sociodemographic block followed by three attitudinal dimensions: cognitive, behavioural, and affective. The cognitive and affective domains—addressing pharmacists’ conceptual understanding and emotional perceptions, respectively—were evaluated using a five-point Likert scale ranging from strongly disagree to strongly agree. The behavioural domains included both short, closed-ended questions and multiple-selection items.

The previous study, reporting the EFA results, found for the affective domain a three-factor structure consisting of Process, Outcome, and Satisfaction [13]. The present study builds upon that work by conducting a CFA to test the hypothesised model.

Given the highly independent and non-correlated nature of the cognitive and behavioural items, EFA and CFA were not applied to these sections of the questionnaire, as they were not expected to reflect latent constructs. These, as well as sociodemographic data, were analysed using descriptive statistics.

Within the scope of this research, recording PIs was defined as documenting the intervention in any format, while classification referred to assigning the intervention to a structured category [17], either using a published framework (e.g., the Act-IP© system [18]) or an in-house developed classification.

### 2.3. Data Analysis

Data were exported in .csv format and analysed using IBM SPSS Statistics (Version 29.0). Descriptive statistics were used to summarise participants’ sociodemographic characteristics, cognitive and behavioural responses. The 5-point Likert scale responses were recoded into two categories—agreement (scores 4–5) and disagreement (scores 1–3)—to simplify the interpretation of response patterns.

The CFA was conducted using IBM Analysis of Moment Structures (AMOS) Software (Version 30) to test the internal structure of the affective domain, based on a hypothesised three-factor model derived from the prior study (i.e., Process, Outcome, and Satisfaction). Nine affective items were included as observed variables.

The model fit was assessed using a range of fit indices: chi-square (χ^2^), Comparative Fit Index (CFI), Tucker–Lewis Index (TLI), Incremental Fit Index (IFI), Normed Fit Index (NFI), Relative Fit Index (RFI), and the Root Mean Square Error of Approximation (RMSEA) with its 90% confidence interval. Standardised regression weights, latent correlations, and variance estimates were examined to evaluate the internal consistency and discriminant validity of the model.

Following the recommendations of Brown [19], a close model fit (PCLOSE) is suggested by values of CFI, TLI, IFI, NFI, and RFI ≥ 0.95, RMSEA ≤ 0.06 with PCLOSE > 0.05, and a non-significant chi-square (χ^2^) (*p* > 0.05), although χ^2^ is known to be highly sensitive to sample size, since larger samples can render even minor model discrepancies statistically significant [20].

Convergent validity was assessed using the Average Variance Extracted (AVE), with values ≥ 0.50 considered acceptable, as per Fornell and Larcker’s criterion [21]. Discriminant validity was examined by comparing the square root of each construct’s AVE with the inter-factor correlations [22].

## 3. Results

### 3.1. Participants’ Background Data

Of the total 1848 registered hospital pharmacists [23], 260 accepted the invitation and completed the questionnaire, corresponding to a 14% net response rate.

The mean age of respondents was 42 years (SD = 10), and the majority were women (90%). The majority of respondents (69.2%) were specialists in hospital pharmacy, and 1.2% were currently enrolled in the hospital pharmacy residency program. In terms of professional experience, 46.5% of respondents had up to 10 years of experience in hospital pharmacy. More experienced pharmacists (i.e., more than 25 years of practice) represented 13.1% of the sample. Regarding the type of institution, most respondents (78.8%) worked in a hospital setting integrated into a Local Health Unit (LHU), an integrated healthcare organization combining hospital and primary care services within a defined geographical area.

Respondents reported involvement in a wide range of hospital pharmacy activities. The most frequently selected areas were clinical pharmacy for inpatients (70.4%), followed by outpatient clinical pharmacy (49.6%) and oncology (46.9%). Respondents were also asked whether they were assigned to or held responsibility for specific clinical services. The most frequently reported services were general medicine (12.3%), oncology/haematology (9.6%), general surgery (9.2%), intensive care units (ICUs) (7.7%), and day hospitals (7.3%). Ten percent of respondents reported not being assigned to any particular service, while 28.5% indicated that this type of clinical service organisation did not apply to their professional context. An overview of the participants’ background characteristics is presented in Table 1.

### 3.2. Confirming the Questionnaire Domains

The majority of respondents (91.2%) reported that performing PIs is part of their routine professional activities. Pharmacists reported performing a median of 20 PIs per month (IQR = 5–35), indicating a highly skewed distribution with a few respondents reporting very high counts. However, despite the reported frequency of interventions, the option “never” was the most frequently selected response regarding the recording (28.8%), classification (56.2%), and analysis (52.3%) of PIs. Assuming a total of 100 potential PIs in daily practice, participants estimated that 31.8% of these are not performed, 63.0% are not registered, 76.8% are not classified, and 78.4% are not subjected to any form of analysis.

The most commonly used formats for documenting PIs were Microsoft Excel (42.7%), Computerised Physician Order Entry (CPOE) systems (37.3%), and Electronic Health Records (EHRs) (26.5%). Formats such as Google Forms (10.8%), Microsoft Access (9.2%), and paper-based records (11.0%) were reported less frequently. Concerning the classification of PIs, slightly more than half (55.8%) of the respondents reported that, in their institutions, PIs were not subject to any classification. Only 2.7% of the participants reported using a validated classification, whereas 41.5% use an in-house developed classification without further validation. Of the seven participants who reported using a published, valid classification, one used the Faus–Dáder classification [12], one the CLEO tool [13], one the Pharmaceutical Care Network Europe (PCNE) framework [14], and the other four were unable to specify the classification used. These results are compiled in Table 2.

The results for the cognitive and affective domains are summarised in Table 3.

### 3.3. Confirmatory Factor Analysis

The three-factor CFA model demonstrated an overall acceptable fit. Although the chi-square was statistically significant, χ^2^ (24) = 42.011, *p* = 0.013, this result was expected given the sample size. Other fit indices indicated good model adequacy: CFI = 0.979; TLI = 0.968; IFI = 0.979; NFI = 0.953; RFI = 0.929; RMSEA = 0.054 (90% CI: 0.038–0.065); PCLOSE = 0.378. The Process factor displayed strong and consistent standardised loadings, ranging from 0.648 (PRO1) to 0.823 (PRO4), with PRO2 (0.693) and PRO3 (0.711) also showing adequate contributions, supporting its internal coherence. Its Average Variance Extracted (AVE) was 0.521, meeting the recommended threshold.

The Outcome factor showed heterogeneous contributions: OUT2 loaded strongly (0.814), whereas OUT1 (0.406) and OUT3 (0.402) presented weaker associations. The AVE was 0.330, below the recommended threshold, indicating limited convergent validity.

The Satisfaction factor presented estimation anomalies, with SAT1 displaying an excessively high standardised loading (2.706) and SAT2 a very low value (0.293), accompanied by a negative error variance (Heywood case). Due to these anomalies, the AVE could not be meaningfully interpreted.

Discriminant validity was examined using the Fornell–Larcker criterion. The square root of the AVE for Process (√0.521 = 0.72) and Outcome (√0.330 = 0.57) were both lower than their latent correlation (r = 0.955), indicating a lack of discriminant validity between these constructs. In contrast, Satisfaction showed negligible correlations with Process (r = 0.027) and Outcome (r = 0.009); however, due to the estimation anomalies and inadmissible AVE, discriminant validity could not be meaningfully evaluated for this factor.

**Figure 1:**
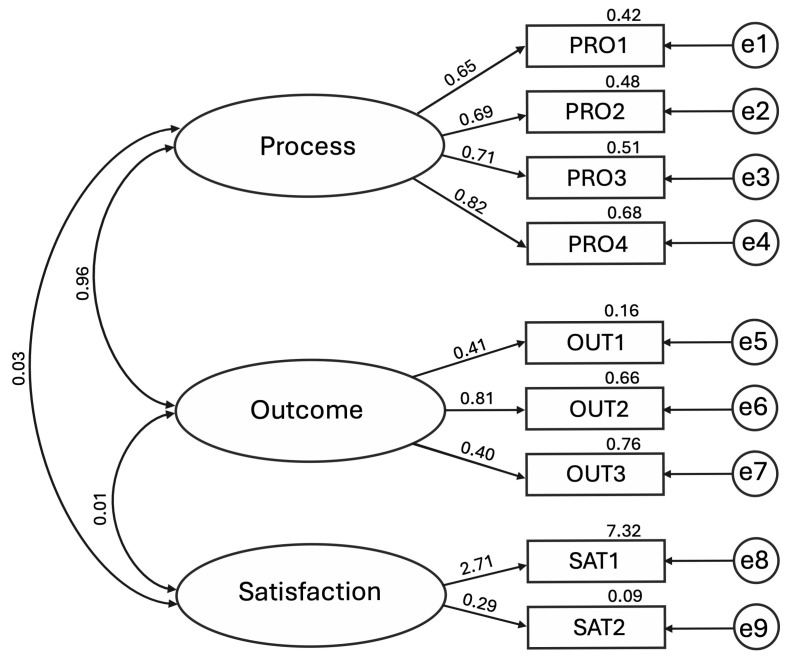
Confirmatory factor analysis of a hypothetical three-factor model of hospital pharmacists’ attitudes towards documenting and classifying pharmaceutical interventions (N = 260); latent constructs: Process (PRO), Outcome (OUT), and Satisfaction (SAT); observed variables: PRO1–PRO4, OUT1–OUT3, and SAT1–SAT3 correspond to the questionnaire items retained in the final model.

A graphical representation of the model is provided in Figure 1.

Detailed unstandardised and standardised regression weights, AVE values, and inter-factor correlations are presented in Table 4.

## 4. Discussion

This study builds on previous research that focused on the pretest, factor structure, validity, and reliability of a novel tool designed to assess pharmacists’ perspectives and routines in documenting and classifying PIs within hospital settings [13]. However, further validation and assessment of its broader impact were needed, which was pursued through the nationwide application of the questionnaire, while recognising that the sample was not designed to guarantee statistical representativeness or power for broader generalisation.

The questionnaire, targeting individual hospital pharmacists in Portugal, obtained a 14% response rate. Despite reminders and a user-friendly platform, participation remained modest, though consistent with previous pharmacist surveys in Portugal, which are scarce and typically report rates around 11–12% [30]. At the European level, hospital pharmacy surveys such as the EAHP Statements Survey achieved 14% in 2018 and 19% in a later edition, although the Portuguese rate in that study was only 10% [31,32]. Thus, our findings align with international evidence of limited engagement in this professional group. While modest, a 14% response rate can be considered acceptable for voluntary, non-incentivised surveys of healthcare professionals, where survey fatigue is a recognised challenge [33]. Nonetheless, concerns about representativeness remain. Portugal lacks updated sociodemographic data on hospital pharmacists, and the only available indicator—the proportion of pharmacists with the Hospital Pharmacy specialty (51.2%)—is lower than observed in our sample (69.2%), [23] suggesting possible overrepresentation of specialists with greater interest in the survey topic.

Portuguese pharmacists reported performing an average of 49 PIs per month (SD = 196), a value higher than that described in studies conducted in other countries, where monthly averages range between 20 and 30 PIs [34,35,36]. These latter studies were retrospective and relied on documented interventions, whereas in our study, the data are self-reported and therefore subject to self-reporting bias. A substantial proportion of pharmacists acknowledged that they never record (28.8%), classify (56.2%), or analyse (52.3%) the PIs performed, and a similar pattern emerged when considering all potential PIs (100%). These findings may be explained by dissatisfaction with current documentation and classification systems and by the frequent use of non-validated, in-house classifications, which limits the generalisability and utilisation of data. For instance, only 7% of the respondents reported using a validated classification, and, even within this subgroup, there was heterogeneity, with some applying frameworks centred on DRPs, such as PCNE, or the Faus-Dáder model, originally developed for the provision of pharmaceutical care. Taken together with broader challenges faced globally by hospital pharmacy services, such as lack of time, limited staff, and increasing workload pressures [37,38], these factors help explain the low rate of PI documentation and classification reported.

Within the cognitive domain, respondents demonstrated high levels of agreement with the four literature-based definitions of PIs [16,17,18,19]. Despite differences in wording and emphasis, these definitions consistently reflect core concepts such as the prevention and resolution of DRPs, interprofessional collaboration, and the optimisation of medication use and therapeutic outcomes, indicating a strong conceptual alignment among hospital pharmacists. Item (e), which did not present a definition but instead addressed the potential ambiguity of the term ‘intervention’ when interpreted by non-pharmacist stakeholders [28], received a slightly lower agreement rate (86.2%). This result may reflect a perceived need to increase awareness of the scope and nature of hospital pharmacists’ clinical roles, particularly among healthcare administrators and other professional groups.

Previous studies have underscored the importance of clearly communicating pharmacists’ contributions to patient safety, therapeutic optimisation, and interdisciplinary care, as a way to support their effective integration within healthcare teams [39,40,41,42]. Strengthening the articulation of this professional identity may also facilitate more structured practice-based research, contribute to broader recognition of the pharmacist’s role. However, only 53.1% of respondents agreed with the statement “There is no internationally recognised and widely accepted definition of PI.” This finding indicates that, while formal international consensus is indeed lacking, the strong conceptual convergence across the four literature-based definitions in this study appears to foster a shared understanding among hospital pharmacists. In practice, this internal consistency may lead professionals to perceive existing definitions as sufficiently robust for clinical application—even without official harmonisation.

Respondents expressed positive attitudes towards PIs, as evidenced by the high level of agreement on the need to classify and record interventions, to standardise classification systems, to assess their impact, and on the notion that the pharmacist’s presence in the clinical setting can increase the number of PIs performed. All respondents agreed that PIs contribute positively to health outcomes, a finding extensively supported in the literature [1,43,44,45]. Nevertheless, there was a substantial level of dissatisfaction with the systems currently used to record and classify PIs, highlighting an unmet need within Portuguese hospital pharmacy practice.

The affective domain of the tool, originally conceptualised with three subdimensions (Process, Outcome, and Satisfaction), demonstrated overall adequate psychometric properties in previous exploratory analyses [13]. In the present study, CFA was conducted to test its structural validity and goodness of fit. The analysis provided partial support for the hypothesised three-factor structure: while the overall model fit indices indicated adequacy, a closer inspection of standardised regression weights, AVE values, and latent correlations revealed important psychometric limitations.

The Process dimension emerged as the most stable and interpretable factor. All items demonstrated strong and statistically significant standardised regression weights, and the construct achieved an AVE above the recommended threshold (0.52), supporting adequate convergent validity. This suggests that pharmacists clearly recognise and differentiate procedural elements related to PI documentation, including classification standards and documentation protocols. In contrast, the Outcome factor presented substantial challenges. Although OUT2 displayed a strong loading, OUT1 and OUT3 had much weaker contributions. Moreover, this factor showed very high correlation with Process (r = 0.955), failing to meet the Fornell–Larcker criterion and indicating a lack of discriminant validity and potential redundancy between the two constructs. This finding may reflect a real-world perception among pharmacists that procedural rigour and clinical outcomes are closely intertwined in documentation practices, raising the question of whether these should be treated as distinct latent dimensions.

The Satisfaction factor proved psychometrically unstable. One item (SAT1) produced an inadmissible loading (Heywood case), while the other (SAT2) had a very low and non-significant loading, leading to AVE values far below acceptable thresholds. This pattern indicates model misspecification and suggests that the construct is not being adequately captured by the current items. A likely explanation is the semantic and spatial proximity of (e.g., SAT1 and SAT2) in the questionnaire layout, which may have induced redundancy or response bias. Respondents may have interpreted these items similarly despite their intended conceptual distinction. This suggests the need for item reformulation or alternative conceptualisation.

### Strengths and Limitations

The nationwide scope and the application of CFA allowed for the assessment of the structural properties of the tool, providing partial support for the hypothesised model. The Process domain demonstrated coherence and convergence validity, suggesting potential utility in practice evaluation, research, and policy. By contrast, the Outcome factor showed insufficient discriminant validity due to its high overlap with Process, and the Satisfaction factor revealed problematic item performance and estimation anomalies, indicating that both domains require further refinement.

Additional limitations should be acknowledged. As mentioned earlier, although the national application expanded the scope of testing, the sample was not designed to ensure statistical representativeness or power for broader generalisation. In addition, the low response rate may have reduced statistical power, reliability, and precision, while the reliance on self-reported data introduces the possibility of reporting bias. The predominance of specialist and more experienced pharmacists among respondents may also have influenced the attitudinal profile observed, limiting generalizability to less experienced professionals. The limited number of items per factor, defined based on evidence from the literature and the previous EFA, may also have affected model stability and some reliability indicators. To enhance construct validity, future versions of the instrument should consider rewording and spatially separating the Satisfaction items, but also expanding the overall number of items per construct. This would allow a more stable factor structure and stronger reliability estimates. In particular, additional items could better capture the multidimensional nature of professional satisfaction, including perceived usability, relevance to practice, and alignment with professional values. Given the high empirical overlap between Process and Outcome, future modelling could explore representing them as a single latent factor, or as first-order subdimensions under a second-order construct reflecting the perceived utility of PI documentation systems. Despite these limitations, the CFA results support the structural coherence of the Process dimension and provide valuable insights for instrument refinement. These findings contribute to the development of more valid and reliable tools for assessing pharmacists’ affective engagement with documentation practices in clinical settings.

## 5. Conclusions

This study advances the validation of a questionnaire designed to assess hospital pharmacists’ opinions and practices regarding the documentation and classification of PIs. The CFA findings provided evidence for the structural coherence of the Process factor, while highlighting overlap between domains and weaknesses in Satisfaction items, indicating that further refinement is needed. A discrepancy emerged between theory—reflected in pharmacists’ positive perceptions of the importance of documenting and classifying PIs—and practice, evidenced by their limited engagement in these activities. Although national data suggest strong professional commitment to PIs, they also highlight significant barriers to systematic documentation. Persistent challenges, including dissatisfaction with current systems, lack of standardisation, and workload constraints, underscore the need for targeted interventions to improve PI recording and classification, namely the establishment of a framework enabling data collection and integration from multiple sites, and the creation of a unified system for PI documentation and classification to generate representative national overviews. A future refined tool may provide a contextually relevant resource to support practice evaluation, guide research, and inform policy decisions aimed at strengthening the quality, visibility, and impact of pharmaceutical care in hospital settings.

## Figures and Tables

**Table 1:** Participants’ background data.

**Sociodemographics**	**N (n)**
**Age** (mean (SD))	42 (10)
**Gender**	
Women	234 (90.0)
Man	26 (10.0)
**Hospital Pharmacy Specialist**	
Yes	180 (69.2)
No	80 (30.8)
**Education Level**	
BSc	43 (16.5)
MPharm	145 (55.8)
Postgraduate	31 (11.9)
MSc	34 (13.1)
PhD	7 (2.7)
**Years Working in Hospital Pharmacy**	
≤5	69 (26.5)
6–10	52 (20.0)
11–15	39 (15.0)
16–20	35 (13.5)
20–25	31 (11.9)
>25	34 (13.1)
**Institution Type**	
Hospital—Local Health Unit	205 (78.8)
Primary Care—Local Health Unit	4 (1.5)
Private Hospital	41 (15.8)
Long Term Care Facilities (LTCFs)	10 (3.8)
**Beds Number**	
<100	29 (11.2)
100–250	47 (18.1)
251–500	91 (37.3)
501–1000	53 (20.4)
>1000	31 (11.9)
Not applicable	3 (1.2)
**Hospital Pharmacy Area** (multiple selection allowed)	
Clinical Pharmacy—Inpatients	183 (70.4)
Clinical Pharmacy—Outpatients	129 (49.6)
Oncology	122 (46.9)
Compounding	99 (38.1)
Management	95 (36.5)
Acquisitions	86 (33.1)
Pharmaceutical Consultation	67 (25.8)
Pharmacokinetics	66 (25.4)
Clinical Trials	52 (20.0)
Pharmacy Residency Program	3 (1.2)
Other	19 (7.3)
**Clinical Service Assigned** (multiple selection allowed)	
General Medicine	32 (12.3)
Oncology/Haematology	25 (9.6)
General Surgery	24 (9.2)
ICUs	20 (7.7)
Day Hospitals	19 (7.3)
Paediatrics	16 (6.2)
Emergency	13 (5.0)
Obstetrics & Gynaecology	11 (4.2)
Orthopaedics	10 (3.8)
Nephrology	9 (3.5)
Cardiology	8 (3.1)
Infectious Diseases	6 (2.3)
Neurology	6 (2.3)
Operating Room	6 (2.3)
Other	48 (18.5)
Not assigned to any particular service	26 (10)
Not applicable	74 (28.5)

**Table 2:** PI documentation and classification methods employed (N = 260).

	N (n)
**PI Documentation Methods**	
Microsoft Excel	111 (42.7)
CPOE	97 (37.3)
EHR	69 (26.5)
Google Forms	28 (10.8)
Paper	29 (11.2)
Microsoft Access	24 (9.2)
Other	30 (11.5)
PIs are NOT recorded	18 (6.9)
**PI Classification Methods**	
In-house developed classification	108 (41.5)
Validated Classification	7 (2.7)
▪ Faus-Dáder	1 (0.4)
▪ PCNE	1 (0.4)
▪ CLEO	1 (0.4)
▪ Not able to specify	4 (1.5)
PIs are NOT classified	145 (55.8)

**Table 3:** Results for cognitive and affective domains (N = 260).

		Level of Agreement n (%)	Level of Disagreement n (%)
	**Cognitive Items**		
	(a) A pharmacist intervention (PI) may be defined as an act or action that prevents medication therapy problems and optimises drug therapy for individual patients in cooperation with other healthcare professionals [24].	253 (97.3)	7 (2.7)
	(b) PIs can be defined as any professional activity by the pharmacist directed towards improving the quality use of medicines and resulting in a recommendation for a change in the patient’s medication therapy, means of administration, or medication-taking behaviour [25].	238 (91.5)	22 (8.5)
	(c) PIs encompass all activities relating to safe medication utilisation and optimising patient therapeutic outcomes in conjunction with other healthcare professionals, ultimately improving patient management or therapy [26].	245 (94.5)	15 (5.8)
	(d) PI is defined as “any communication/action solving and/or avoiding drug-related problems (DRPs)” and includes the “management of existing DRPs as well as any proactive approach avoiding potential DRPs within the medication use process [27].	248 (95.4)	12 (4.6)
	(e) Although the definition of intervention is well understood by pharmacist vernacular, its scope of interpretation may be ambiguous to other healthcare providers and hospital administrators [28].	224 (86.2)	36 (13.8)
	(f) There is no internationally recognised and widely accepted definition of PI [29] by most hospital pharmacists.	138 (53.1)	122 (46.9)
	**Affective Items**		
**Process**	1. I believe that PIs should be classified in a standardised way across all hospital institutions.	242 (93.1)	18 (6.9)
	2. The recording of PIs should be mandatory	202 (77.7)	59 (22.3)
	3. I consider it necessary to classify PIs	239 (91.9)	21 (8.1)
	4. I consider it important to record PIs	250 (96.2)	10 (3.8)
**Outcome**	5. PIs contribute positively to health outcomes	260 (100)	0 (0.0)
	6. I consider it important to evaluate the impact of PIs	253 (97.3)	7 (2.7)
	7. I believe that the presence of the pharmacist in the clinical service can increase the number of PIs performed.	251 (96.5)	9 (3.5)
**Satisfaction**	8. I am satisfied with the system currently used in my institution for **recording** PIs	61 (23.5)	199 (76.5)
	9. I am satisfied with the system currently used in my institution for **classifying** PIs.	38 (14.6)	222 (85.4)

**Table 4:** Confirmatory factor analysis of a hypothetical three-factor model (N = 260).

Factor	Item	SRW	URW	SE	AVE
**Process**	PRO1	0.648	1.000	–	
	PRO2	0.693	1.408	0.151	
	PRO3	0.711	1.074	0.113	
	PRO4	0.823	1.154	0.109	0.52
**Outcome**	OUT1	0.406	1.000	–	
	OUT2	0.814	2.995	0.493	
	OUT3	0.402	1.653	0.359	0.33
**Satisfaction**	SAT1	2.706 ^†^	1.000	–	
	SAT2	0.293	0.100	0.672	n.a.

† Heywood case (standardised loading > 1). PRO—Process; OUT—Outcome; SAT—Satisfaction; URW—unstandardised regression weight; SRW—standardised regression weight; SE—standard error of URW; AVE—Average Variance Extracted. Inter-factor correlations; Process ⟷ Outcome = 0.955; Process ⟷ Satisfaction = 0.027; Outcome ⟷ Satisfaction = 0.009.

## Data Availability

The data presented in this study are available on reasonable request from the corresponding author due to privacy and confidentiality considerations and institutional data protection policies.

## Supplementary Materials

The following supporting information can be downloaded at: https://www.mdpi.com/article/10.3390/pharmacy13060159/s1, File S1: English translation of the original questionnaire (Practices of Recording and Classifying Hospital Pharmaceutical Interventions in Portugal).

## Abbreviations

The following abbreviations are used in this manuscript:

AMOS	Analysis of Moment Structures
AVE	Average Variance Extracted
CFA	Confirmatory Factor Analysis
CFI	Comparative Fit Index
CPOE	Computerised Physician Order Entry
DRP	Drug-Related Problem
EAHP	European Association of Hospital Pharmacists
EFA	Exploratory Factor Analysis
EHR	Electronic Health Record
ICU	Intensive Care Unit
IFI	Incremental Fit Index
LHU	Local Health Unit
LTCFs	Long-Term Care Facilities
NFI	Normed Fit Index
OUT	Outcome (Latent Factor)
PCLOSE	P of Close Model Fit
PCNE	Pharmaceutical Care Network Europe
PI	Pharmacist Intervention
PRO	Process (Latent Factor)
RFI	Relative Fit Index
RMSEA	Root Mean Square Error of Approximation
SAT	Satisfaction (Latent Factor)
SD	Standard Deviation
SE	Standard Error of Unstandardised Regression Weights
SRW	Standardised Regression Weights
TLI	Tucker–Lewis Index
URW	Unstandardised Regression Weights
χ^2^	Chi-square
