# Supplementary material for: Hospital Pharmacists’ Perspectives on Documenting and Classifying Pharmaceutical Interventions: A Nationwide Validation Study in Portugal
Source: Pharmacy (Basel). 2025 Nov 1;13(6):159. doi: 10.3390/pharmacy13060159 (PMC12641766; doi:10.3390/pharmacy13060159)
Supplement: Supplementary file 1 [file pharmacy-13-00159-s001.zip › pharmacy-3914153-supplementary.pdf]

Practices of Recording and Classifying Hospital Pharmaceutical Interventions in Portugal

Dear Colleague,

This questionnaire is part of a study conducted as part of a Doctoral Project in Pharmacy at the Faculty of Pharmacy, University of Lisbon (FFUL). The study aims to evaluate the practices of documentation, analysis, and classification of pharmaceutical interventions in hospital settings in Portugal. Therefore, it should only be answered by pharmacists professionally practicing in the field of hospital pharmacy.

Your participation in this study is anonymous, and we kindly request that you answer all questions spontaneously and sincerely. Completing the questionnaire will take less than 15 minutes.

Your participation in this study is voluntary. You have the right to withdraw from the study at any time without providing a reason, i.e., without having to justify your decision, and without any present or future consequences. The data will remain under the custody of the principal investigator and will be destroyed after five years or upon request by the participants. Participation in this study incurs no associated costs.

In accordance with the applicable legislation and the rules of the National Data Protection Commission (CNPD) (Deliberation no. 1704/2015, of October 22, and Decree-Law no. 67/1998, of March 18), and as required by the General Data Protection Regulation (GDPR; Regulation (EU) 2016/679 of the European Parliament and the Council, of April 27, 2016), the confidentiality of all responses is guaranteed. The data collected as part of this study will only be statistically processed by the research team for scientific research purposes and based strictly on what is necessary to address the study's objective. This study was previously approved by the Human Research Ethics Committee (CEISH), FFUL, under reference 01/2024.

If you need more information about the study and/or your rights, you may contact the principal investigator through the following details:
Prof. Afonso Miguel das Neves Cavaco
FFUL, Av. Prof. Gama Pinto s/n, 1649-003, Lisbon
(Phone: 217946456, Email: acavaco@ff.ulisboa.pt)

INFORMED CONSENT

Are you willing to participate in this study based on the described objectives?
By selecting "Yes" and proceeding to the next page, you agree to the processing of the provided information for the purposes described above.

- (●) YES, I wish to participate in the study and confirm my consent for the collection, retention, and use of my personal data as described above.
- ( ) NO, I do not wish to participate in the study.

1. What is your year of birth?

____

2. What is your gender?

- ( ) Female
- ( ) Male
- ( ) Non-binary
- ( ) Prefer not to say

3. What is your level of academic qualification?

- ( ) Pre-Bologna BSc
- ( ) MPharm
- ( ) Postgraduate
- ( ) MSc
- ( ) PhD

4. Are you a specialist in Hospital Pharmacy?

- ◯ Yes
- ◯ No

5. How many years have you been working in Hospital Pharmacy?

- ◯ Less than 5 years
- ◯ Between 5 and 10 years
- ◯ Between 11 and 20 years
- ◯ Between 21 and 30 years
- ◯ More than 30 years

6. In which area(s) of Hospital Pharmacy do you practice?

- ☐ Inpatients Drug Distribution
- ☐ Outpatients Drug Distribution
- ☐ Oncology Pharmacy
- ☐ Pharmaceutical Consultation
- ☐ Compounding
- ☐ Pharmacokinetics
- ☐ Clinical Trials
- ☐ Acquisitions and Management
- ☐ Other

Which?

7. What type of institution do you currently work in?

- ◯ Hospital - Local Health Unit
- ◯ Primary Care - Local Health Unit
- ◯ Military Hospital
- ◯ Private Hospital
- ◯ Long-Term Care Facility

8. How many beds does the institution where you currently work have?

- ◯ Less than 100 beds
- ◯ Between 100-250 beds
- ◯ Between 251-500 beds
- ◯ Between 501-1000 beds
- ◯ More than 1000 beds
- ◯ Not applicable

9. If you are assigned to one or more clinical services, please specify which ones.

10. For each of the following statements, select the option that best reflects your opinion. References

	Strongly disagree	Disagree	Neither agree nor disagree	Agree	Strongly Agree
a) A pharmacist intervention (PI) may be defined as an act or action that prevents medication therapy problems and optimise drug therapy for individual patients in cooperation with other healthcare professionals. (1)	◯	◯	◯	◯	◯
b) PI can be defined as any professional activity by the pharmacist directed towards improving the quality use of medicines and resulting in a recommendation for a change in the patient's medication therapy, means of administration or medication-taking behaviour. (2)	◯	◯	◯	◯	◯
c) PI encompasses all activities relating to safe medication utilisation and optimising patient therapeutic outcomes in conjunction with other health care professionals, ultimately improving patient management or therapy. (3)	◯	◯	◯	◯	◯
d) PI is defined as any communication/action solving and/or avoiding drug-related problems (DRPs) and includes the management of existing DRPs as well as any proactive approach avoiding potential DRPs within the medication use process. (4)	◯	◯	◯	◯	◯
e) Although the definition of PI is well understood by pharmacist vernacular, its scope of interpretation may be ambiguous to other healthcare providers and hospital administrators. (5)	◯	◯	◯	◯	◯
f) There is no internationally recognized definition of PI (5) and widely accepted by the majority of hospital pharmacists.	◯	◯	◯	◯	◯

11. Is performing PIs part of your daily activities?

◯ Yes

◯ No

12. On average, how many PIs do you perform monthly?

____

13. Select, from the options below, the frequency with which you perform...

	Never	Bimonthly or superior	Monthly	Biweekly	Weekly	2 or more times per week	Daily
a) PIs							
b) Recording of PIs							
c) Classifying PIs							
d) Analysing PIs							

14. Considering **all potential pharmaceutical interventions (100%)**, in practice, to what extent (X%) do you...

	Never (0%)	Always (100%)
a) Perform PIs		
b) Record PIs		
c) Classify PIs		
d) Analyse PIs		

15. On what medium are PIs recorded in your institution?

- [ ] Microsoft Access
- [ ] Microsoft Excel
- [ ] Google Forms
- [ ] Computerized Physician Order Entry (CPOE) system
- [ ] Electronic Health Record
- [ ] Paper
- [ ] Other

Which?

16. In your institution, PIs are classified according to:

- ○ In-house developed classification
- ○ Validated classification (e.g., Act-IP®)

Which?

- ○ PIs are not classified

17. For each of the following statements, select the option that best reflects your opinion.

	Strongly disagree	Disagree	Neither agree nor disagree	Agree	Strongly Agree
a) IFs contribute positively to health outcomes.	○	○	○	○	○
b) I consider it important to **record** the PIs.	○	○	○	○	○
c) The **recording**of PIs should be mandatory.	○	○	○	○	○
d) I consider it important to **classify** the IFs.	○	○	○	○	○
e)I believe that IFs should be **classified** in a standardized way in all hospital institutions.	○	○	○	○	○
f)I consider it important to **evaluate** the impact of IFs.	○	○	○	○	○
g) I believe that the presence of a pharmacist in the ward can increase the number of PIs performed.	○	○	○	○	○
j) EI'm satisfied with the system currently used in my institution for **recording** PIs.	○	○	○	○	○
k)I'm satisfied with the system currently used in my institution for **classifying** PIs.	○	○	○	○	○

Thank you very much for your participation!

If you have any comments, observations, or suggestions, you may use the field below:

If you are interested in receiving information about the study results later, please provide your email address below.
